# Molecular cytogenetic analysis of partial monosomy 10p and trisomy 10q resulting from familial pericentric inversion (10): a first case report in Chinese population

**DOI:** 10.1186/s13039-022-00599-w

**Published:** 2022-06-07

**Authors:** Jianlong Zhuang, Chunnuan Chen, Rongfu Huang, Qi Luo, Yuying Jiang, Shuhong Zeng, Yuanbai Wang, Yingjun Xie

**Affiliations:** 1 Center for Prenatal Diagnosis, Quanzhou Women’s and Children’s Hospital, Quanzhou, 362000 People’s Republic of China; 2 Department of Neurology, The Second Affiliated Hospital of Fujian Medical University, Quanzhou, 362000 People’s Republic of China; 3 Laboratory department, The Second Affiliated Hospital of Fujian Medical University, Quanzhou, 362000 People’s Republic of China; 4 Department of Public Health for Women and Children, Quanzhou Women’s and Children’s Hospital, Quanzhou, 362000 People’s Republic of China; 5 Department of Obstetrics and Gynecology, Guangdong Provincial Key Laboratory of Major Obstetric Diseases, The Third Affiliated Hospital of Guangzhou Medical University, Guangzhou, 510150 People’s Republic of China; 6 Key Laboratory of Reproduction and Genetics of Guangdong Higher Education Institutes, The Third Affiliated Hospital of Guangzhou Medical University, Guangzhou, 510150 People’s Republic of China

**Keywords:** Recurrent spontaneous abortion, Molecular cytogenetics, Monosomy 10p, Trisomy 10q, Pericentric inversion

## Abstract

**Background:**

Chromosome aberrations of 10p monosomy and 10q trisomy resulting from parental pericentric inversion 10 are extremely rare, and to date, very few reports have been published on the matter.

**Case Presentation:**

A 30-year-old pregnant woman with recurrent pregnancy loss is enrolled in this research. In this pregnancy, spontaneous abortion occurred in the first trimester of her pregnancy. Chromosomal microarray analysis of the abortion tissue showed a partial 10p monosomy (arr[GRCh37] 10p15.3p11.21(100,047_34,848,853) × 1) and a duplication of 10q (arr[GRCh37] 10q26.13q26.3(126,093,990_135,426,386) × 3). Further parental karyotype analysis indicated that the chromosomal abnormalities in the fetus was resulted from paternal pericenric inversion inv(10)(p11.21q26.13). This study presents the first case of a large deletion of 10p combined with 10q trisomy, resulting in pregnancy loss. Of these two manifestations, the large deletion of chromosome 10p may be the primary reason for spontaneous abortion in this subject.

**Conclusions:**

This study presents the first case of partial 10p monosomy associated with 10q trisomy in Chinese population. It provides more information on the chromosome aberration of 10p monosomy and 10q trisomy and further strengthens the application value of microarray in the molecular etiological diagnosis of recurrent spontaneous abortion.

## Background

Novel genetic abnormalities in the fetus and parental inheritance are a common cause of accidental or recurrent spontaneous abortion (RSA). These irregularities include fetal aneuploidy abnormalities, copy number variants, single-gene diseases, etc. [1] However, the most common parental inheritance factors are the balanced translocation of parental chromosomes, or Robertson translocation and inversion. In RSA cases, 5% of parents have chromosomal rearrangements, including balanced translocations, and Robertson translocations and inversions [2]. In recent years, chromosomal microarray technology has gradually replaced karyotype analysis in the genetic analysis of spontaneous abortion, with advantage of automatic and easily identification of chromosomal abnormalities, as well as increased detection rate of chromosomal abnormalities without cell culture. [3, 4]

Terminal chromosomal duplication and deletion that occurs in the same chromosome is usually the result of parental inversion. Chromosomal pericentric inversion commonly forms a unique inversion loop in the first meiosis, resulting in four different gametes, including a normal chromosome and an inverted chromosome. It also causes two kinds of chromosomal recombinants with deletions and duplications of distal regions [5]. In general, the smaller of the inverted segment, the larger of the duplication and deletion segments in the gametes. This consequently results in a tendency towards infertility, miscarriage, and stillbirth. This chromosomal rearrangement also leads to the production of unbalanced gametes including 10p monosomy associated with 10q trisomy and opposite terminal chromosomal rearrangements. However, few reports are available on the pericentric inversion of chromosome 10.

In this study, we identified a recombinant chromosome l0, which was caused by a paternal pericentric inversion inv(10)(p11.21q26.13). This was the first-ever observation of a large deletion of chromosome 10p associated with 10q trisomy, resulting in spontaneous abortion in the first trimester. Besides, we reviewed the molecular cytogenetics of chromosomal abnormalities resulting from familial pericentric inversion 10 (Table 1).

**Table1:** Molecular cytogenetics findings of recombinant chromosome 10 resulting from familial pericenric inversion

	Dutrillaux et al.[6]	Rodriguez et al.[7]	Ohba et al.[8]	Kulharya et al.[9]			Kozma and Meck [10]		Ciuladaite et al.[5]		Our case
				Patient1	Patient2	Patient3	Patient1	Patient2	Patient1	Patient2	
Age/Sex	Newborn/Male	Newborn /Male	Newborn /Female	Newborn /Male	2.5/Male	Fetus/Female	4/Female	36/Male	13/Female	28/Female	Fetus/Male
Trisomic segment	10q24 → q26	10q24 → qter	10p11.2 → pter	10p11.2 → pter	10p11.2 → pter	10p11.2 → pter	10p11.2 → pter	10p11.2 → pter	10p15.1 → pter	10q26.12 → qter	10q26.13 → qter
Monosomic segment	10p15 → pter	10p15 → pter	10q25.2 → qter	10q26 → qter	10q26 → qter	10q26 → qter	10q26 → qter	10q26 → qter	10q26.12 → qter	10p15.1 → pter	10p11.21 → pter
Breakpoints	10p15-q24	10p15-q24	10p11.2-q25.2	10p11.2-q26	10p11.2-q26	10p11.2-q26	10p11.2-q26	10p11.2-q26	10p15.1-q26.12	10p15.1-q26.12	10p11.21-q26.13
Inheritance	Maternal	Maternal	Maternal	Maternal	Maternal	Maternal	Maternal	Maternal	Maternal	Maternal	Paternal
Clinical phenotypes	Facial deformities, finger deformities, psychomotor development delay, severe hypotonia	Facial deformities, long thin fingers	Facial deformities, clinodactyly of the fifth fingers, clenched hands, club foot, ocular abnormalities	Facial deformities, hypotonic and can not rolled over	Facial deformities, right exotropia, bilateral single palmar creases and fifth finger clinodactyly, absent fifth toenails, moderate motor delay	stillborn occurred after delivery	Facial deformities, increased length of the palm, foot deformities, poor physical growth, severe to profound intellectual disability	Facial deformities, unilateral cleft and corresponding absent tooth, partial hearing loss, and a heart murmur, growth and intellectual disability, increased palm length	Facial deformities, psychomotor and language development delay, strabismus, malocclusion of teeth, tapering fingers, transversal crease in the left palm, deformed feet	Psychomotor development delay, obesity, arched eyebrows, low-set ears, short philtrum	Spontaneous abortion occurred in the first trimester

## Methods

Approximately 2 ml of peripheral blood was collected from both parents. Parental chromosome karyotype analysis was conducted using G-banding technology with approximately 300 bands based on the automatic chromosome harvesting system Sinochrome Chromprep II (Shanghai Lechen Biotechnology Co., Ltd, China). The International System for Human Cytogenomic Nomenclature (ISCN 2020) was used as the reference for delineates karyotypes. The QIAamp DNA Blood Kit (QIAGEN, Germany) was utilized to extract genomic DNA from the abortion tissue and parental peripheral blood of the foetus. SNP array analysis was conducted using Affymetrix CytoScan 750 K, according to the Affymetrix CytoScan Assay user guide (http://www.thermofisher.com). The chromosomal copy number variants were described according to the Human NCBI Build GRCh37 (hg19/2009).

## Case presentation

A 30-year-old pregnant woman (gravida 4, para 0) from Quanzhou City, Fujian province, China came to Quanzhou Women’s and Children’s Hospital because of spontaneous abortion and adverse pregnancy history. Her husband was 31 years old. There was no family history of hereditary disease and the couple denied any consanguinity. At her first pregnancy, termination was conducted at a gestational age of 13^+^ weeks due to nuchal translucency (NT) thickening (7 mm) and fetal oedema. A spontaneous abortion then occurred at 8^+^ weeks during the second pregnancy. The third pregnancy was terminated at gestational age of 11^+^ weeks due to NT thickening (11.2 mm) and hygroma. Unfortunately, the abortion tissues were not available. In the most recent pregnancy, early threatened abortion occurred at gestational age of 8^+2^ weeks of pregnancy, and aborted tissue was discharged at gestational age of 8^+5^ weeks. Molecular investigation was further performed after termination.

**Fig. 1:**
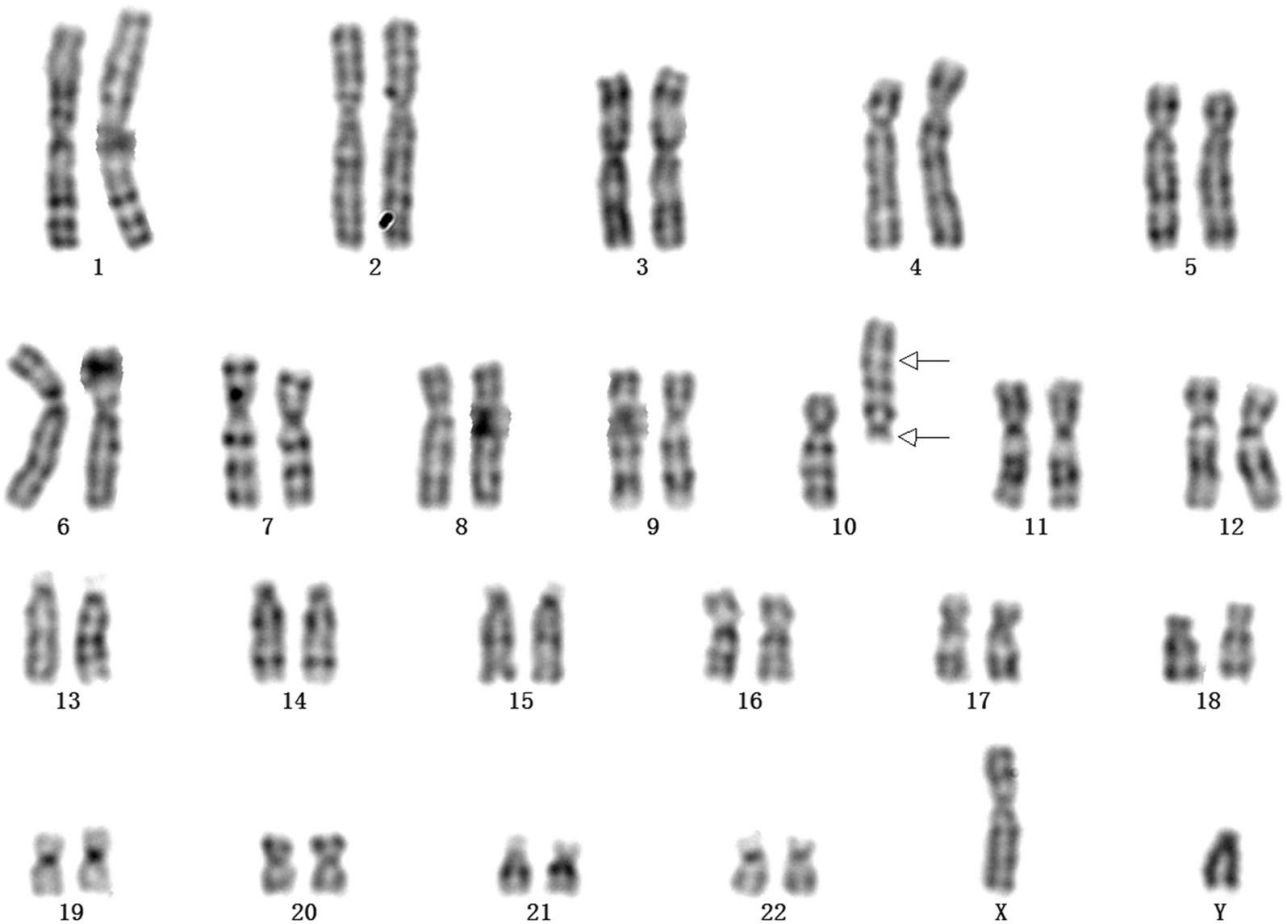
The result of chromosome karyotype in the prospective father. The arrows indicated the breakpoints of inversion of chromosome 10. The upper arrow elicited the breakpoint of 10p11.21 and the lower arrow indicated the location of 10q26.13, and the karyotype of the prospective father was described as 46,XY, inv(10)( p11.21q26.13)

**Fig. 2:**
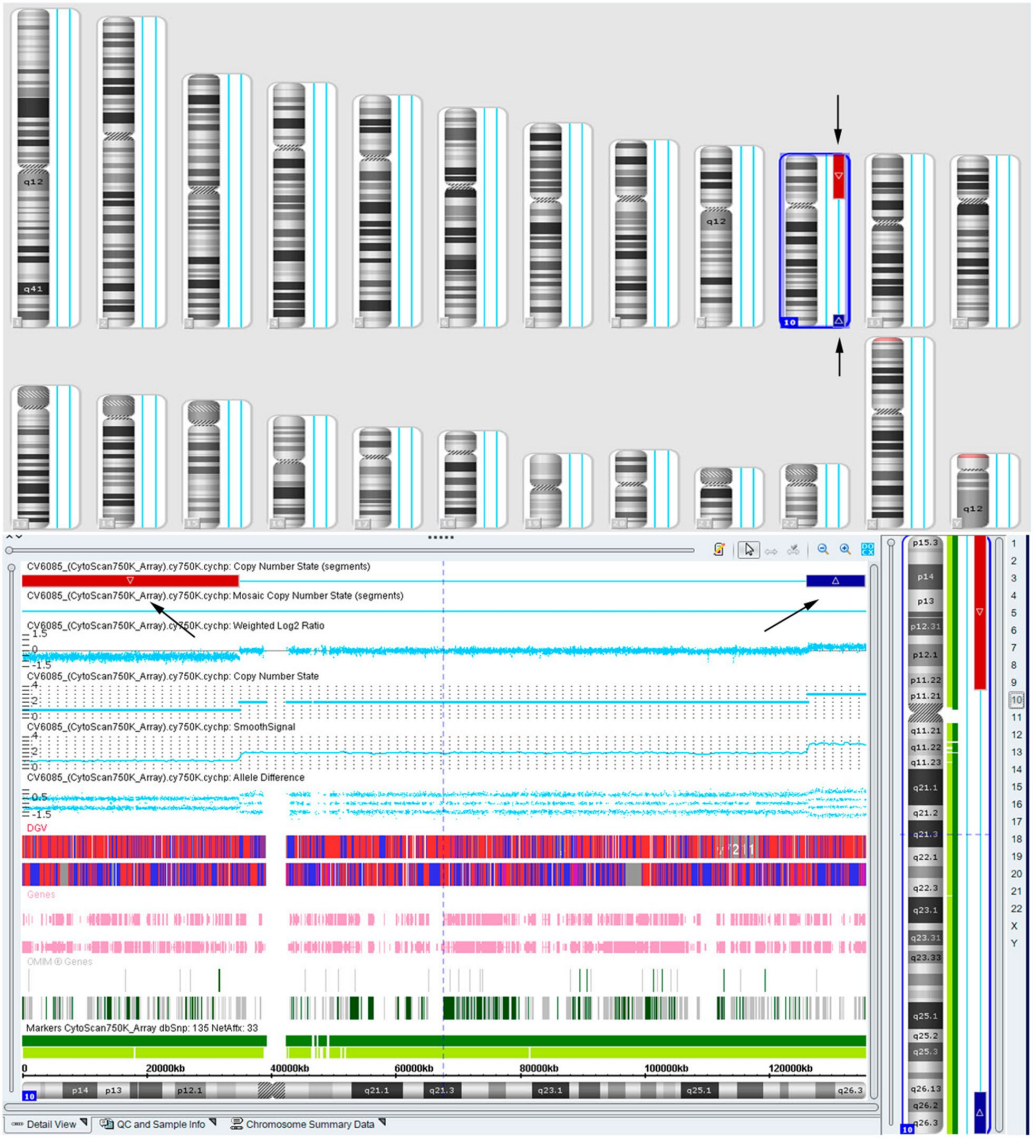
The SNP array detection results of in the fetus. The arrows indicated the locus of duplication and deletion segments. The red bar represents 10p11.21p15.3 deletion and the blue bar indicates the duplication of 10q26.13q26.3

The pregnant woman received prenatal clinical consultation and parental karyotype analysis was further performed. Chromosome karyotype analysis revealed a karyotype of 46,XY,inv(10)(p11.21q26.13) in the prospective father (Fig. 1), and a karyotype of 46,XX in the prospective mother. The SNP array analysis of the abortion tissue showed a 34.7-Mb deletion in 10p11.21p15.3 region (arr[GRCh37] 10p15.3p11.21(100,047_34,848,853) × 1) of chromosome 10 (Fig. 2). Additionally, we observed a 9.3-Mb duplication of chromosome 10q26.13q26.3 region (arr[GRCh37] 10q26.13q26.3(126,093,990_135,426,386) × 3), as illustrated in Fig. 2. The deletion of chromosome 10p11.21p15.3 contained 32 Online Mendelian Inheritance in Man (OMIM) genes and the 10q26.13q26.3 duplication covered 35 OMIM genes. The SNP array was performed in the parents and no copy number variants were observed.

## Discussion and conclusions

Few studies are available on the familial recombination of a pericentric inversion of chromosome 10, a condition which usually results in unbalanced gametes including monosomy 10p associated with trisomy 10q and opposite terminal chromosomal rearrangements. The smaller the inverted segment would lead to more severe phenotype. To our knowledge, this is the first case with large deletion of 10p result from paternal pericentric inversion inv(10)(p11.21q26.13) and leading to spontaneous abortion in the first trimester.

As displayed in Table 1, mostly of the present cases harbored trisomy 10p and monosomy 10q and exhibiting the typical features of trisomy 10p, which was a well-delineated chromosomal abnormality characterization by facial deformities, finger deformities, as well as growth and intellectual disabilities. A previous study conducted by Chen et al. [11] demonstrated a fetus with a 31.65-Mb duplication of 10p15.3p11.22 and a 3.07-Mb deletion of 10q26.3 resulting from paternal pericentric inversion, and the pregnancy was subsequently terminated at gestational age of 23 weeks with the fetus displaying facial dysmorphism of hypertelorism, large low-set ears, a broad and long nasal bridge, a long face, and micrognathia, which were consistent with partial trisomy 10p syndrome.

Recombination in chromosome 10 with partial monosomy 10p and trisomy 10q resulting from parental pericentric inversion is less common. At present, only three cases of partial 10p deletion and 10q duplication resulting from pericentric inversion have been reported in our best knowledge. [5–7] Distal trisomy 10q is a well-recognized syndrome, with typical features including growth retardation, hypotonia, mild to severe intellectual disabilities, and mild to severe psychomotor retardation [12]. Studies revealed that partial 10p deletion and 10q duplication mainly exhibit clinical features of trisomy 10q, which usually present a small deletion in the distal of chromosome 10p [5–7]. However, our case displayed a significant deletion of 10p, which was the first case of a large deletion of chromosome 10p associated with partial trisomy 10q resulting from pericentric inversion. In this study, a deletion fragment range from 10p11.21 to the distal with a 34.7-Mb deletion was identified, which is a rare chromosomal abnormality, and it includes three defined distinct contiguous gene deletion syndromes, including the 10p13-10p14 region contributed to the DiGeorge critical region 2 (DGCR2) syndrome, [13] the 10p14 region containing *GATA3* gene that responsible for hypoparathyroidism, deafness, and renal anomalies (HDR Syndrome), [14] and 10p15.3 microdeletion syndrome [15, 16]. Thus, we believe that the large deletion in chromosome 10p may ascribe to the miscarriage in our study. Since the aborted tissues of the first three pregnancies are not available, the chromosomal abnormality types were unknown. However, a previous study also delineated a fetus who harbored trisomy 10p and monosomy 10q with stillborn after delivery. [9]

In the present study, none of the relevant clinical phenotypes were observed due to early spontaneous abortion. Despite that, the OMIM genes in the 10p monosomy involving *CACNB2*, [17] *KLF6*, [18] *MLLT10*, [19] *RAB18*, [20] *WDR37*, [21] *ZEB1* [22] have been reported to be essential for embryonic critical organs development or neuronal development and migration, which may cause pregnancy loss. While, more work must be done to investigate the critical genes for embryonic development in this region.

In conclusion, we presented a recombinant chromosome l0 resulting from the paternal pericentric inversion inv(10)(p11.21q26.13), which was the first reported case of a large deletion of chromosome 10p associated with 10q trisomy that resulted in spontaneous abortion in the first trimester. Moreover, our study provides more information on the chromosome aberration of 10p monosomy and 10q trisomy and also provides valuable data for prenatal genetic consultation.

## Data Availability

The datasets used and analysed during the current study are available from the corresponding author by reasonable request.

## Abbreviations

SNP array: Single nucleotide polymorphism array
Chr: Chromosome
NT: Nuchal translucency
dup: Duplication
inv: Inversion
rec: Recombinant
RSA: Recurrent spontaneous abortion
OMIM: Online Mendelian inheritance in man
